# Recurrent Chemical Meningitis Due to Parasellar Epidermoid Cyst

**DOI:** 10.7759/cureus.3496

**Published:** 2018-10-25

**Authors:** Bhavesh Trikamji, Mark Morrow

**Affiliations:** 1 Neurology, Harbor University of California Los Angeles Medical Center, Los Angeles, USA

**Keywords:** chemical meningitis, meningitis, meningoencephalitis, epidermoid cyst, sellar lesion

## Abstract

Intracranial epidermoid cysts are exceedingly rare lesions that result from a disorder of gastrulation. They are seen only in the pediatric patient population. We describe a 44-year-old Hispanic woman who presented with acute confusion. The family reported two months of progressive headaches and two weeks of fever, blurred central vision, and restricted visual fields. On examination, the patient appeared ill, with a low-grade fever and stiff neck. Neurological testing was limited but grossly non-focal. Computerized tomography (CT) of the head and magnetic resonance imaging (MRI) of the brain showed a large cystic mass arising in the sella, where it displaced the normal pituitary gland. Cerebrospinal fluid (CSF) showed mildly elevated opening pressure with high protein, low glucose, and neutrophilic pleocytosis. Extensive serum and CSF evaluation were negative for infectious agents. The patient was initially started on empiric treatment for presumed infectious meningoencephalitis. As tests for bacterial and viral pathogens were normal, she was switched to fluconazole. The mental status returned to normal and she was discharged home with close follow up. She returned one month later with a recurrent headache, nausea, and stiff neck. The examination showed meningismus but was otherwise non-focal. MRI of the brain showed no change in the parasellar mass. Repeat CSF showed an even higher white blood cell (WBC) count and protein with continued hypo-glycorrhachia. She underwent trans-nasal trans-sphenoidal hypophysectomy and pathology revealed a squamous epithelium-lined keratin-filled cyst suggestive of an epidermoid cyst. The patient responded well to surgery and was discharged on pituitary hormone supplements alone. To our knowledge, this is a first adult case of recurrent chemical meningitis secondary to a ruptured epidermoid cyst in the sella.

## Introduction

Meningitis is generally of infectious origin and occurs as a monophasic illness; it may be associated with a host of organisms. We present an unusual case of recurrent meningitis associated with a sellar-region epidermoid cyst, thought to be due to the intermittent release of toxic fluid contents. Central nervous system (CNS) epidermoid cysts are rare and only encountered in the pediatric population.

## Case presentation

**Figure 1:**
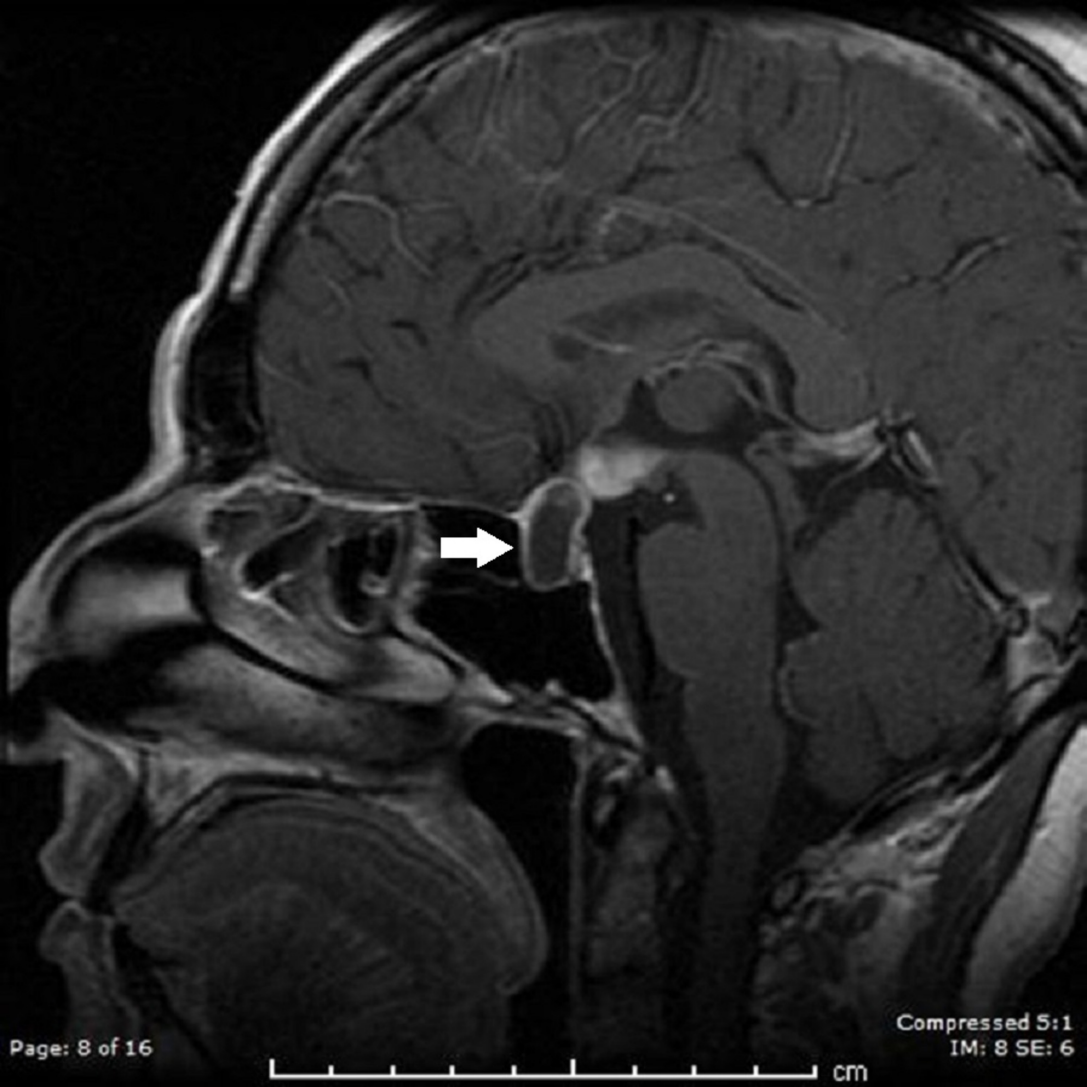
Post-contrast sagittal T1 magnetic resonance imaging on initial admission, showing large parasellar cystic mass

A 44-year-old Hispanic woman presented with acute confusion. The family reported two months of progressive headaches and two weeks of fever, blurred central vision, and restricted visual fields. The review of systems was positive for recent nausea and vomiting. There was a remote history of treatment for latent tuberculosis and a recent history of a treated urinary tract infection. On examination, the patient appeared ill, with a low-grade fever (38.5°C) and stiff neck. She was somnolent and oriented only to self when aroused. Neurological testing was limited but grossly non-focal. A computerized tomography (CT) scan of the head and magnetic resonance imaging (MRI) of the brain showed a large cystic mass arising in the sella, where it displaced the normal pituitary gland. It extended over 1 cm above the tuberculum sella, compressing the optic chiasm (Figure 1). There was considerable contrast enhancement of the cyst wall and of the overlying chiasm and adjacent hypothalamic region. The cerebrospinal fluid (CSF) analysis showed mildly elevated opening pressure (30 cm H2O), with high protein (104) and low glucose (29). There were 835 white blood cells (WBCs, 56% neutrophils). Extensive serum and CSF evaluation were negative for infectious agents. Hormonal assays revealed evidence of pan-hypopituitarism. CT scanning of chest, abdomen, and pelvis was unremarkable for metastatic etiology. The patient was initially started on vancomycin, ceftriaxone, acyclovir, and dexamethasone for presumed infectious meningoencephalitis. As tests for bacterial and viral pathogens were normal, she was switched to fluconazole. The mental status returned to normal and she wished to be discharged home on fluconazole and pituitary hormone replacements.

**Figure 2:**
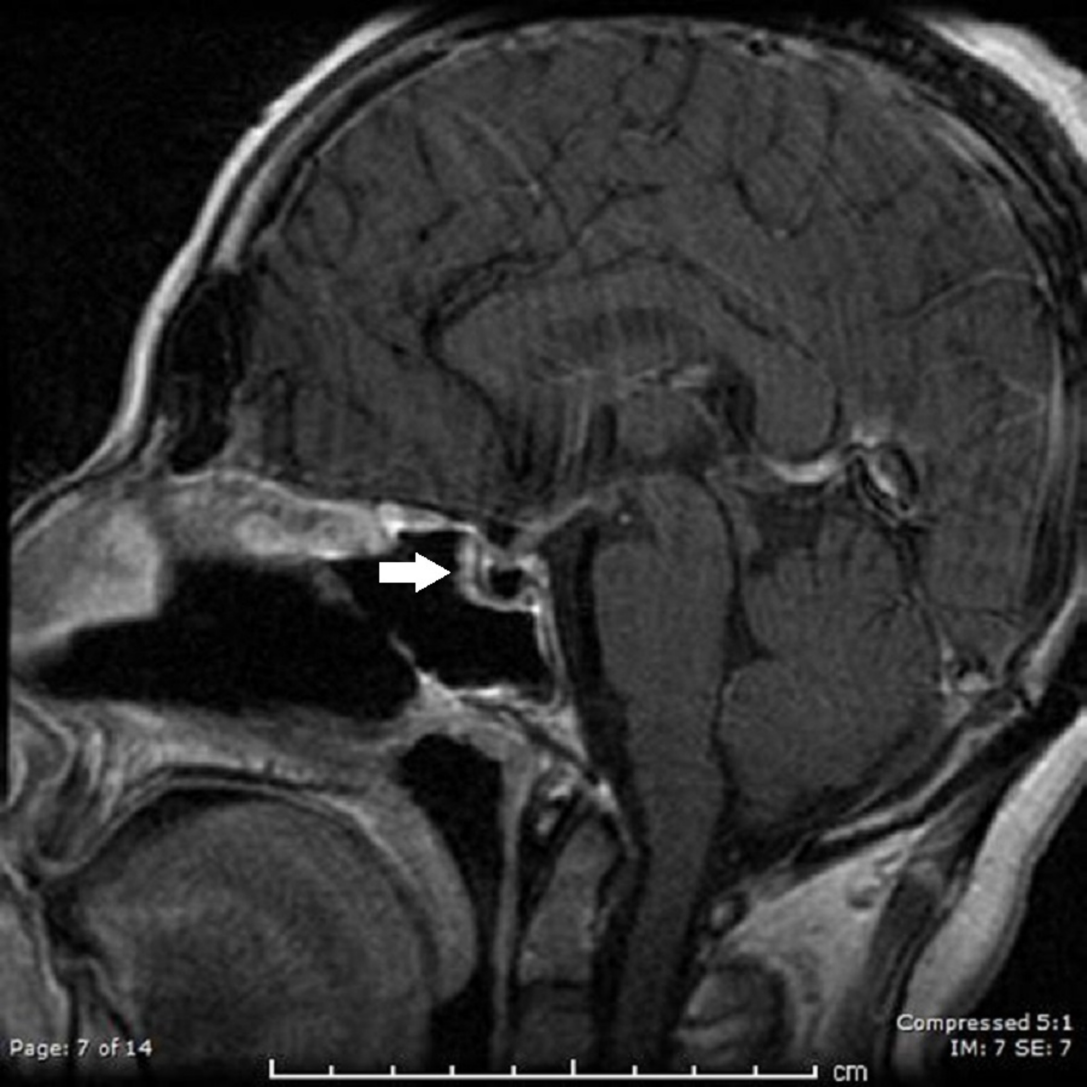
Post-contrast sagittal T1 magnetic resonance imaging after surgical extirpation of cyst

**Figure 3:**
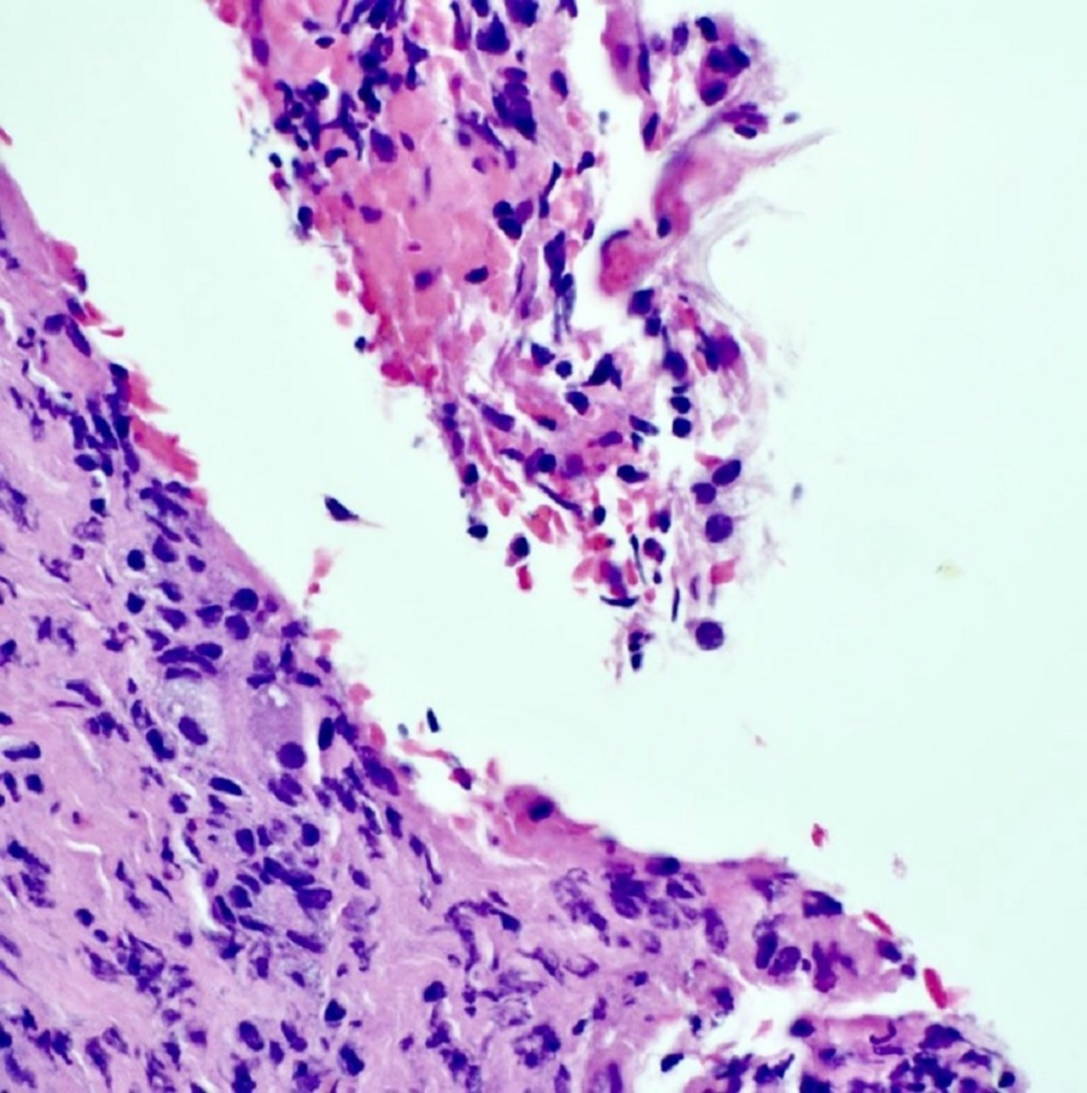
Hemotoxylin and eosin staining of cyst contents showing keratin with surrounding inflammation

She missed her follow-up and returned one month later with a recurrent headache, nausea, and stiff neck. The examination showed meningismus but was otherwise non-focal. MRI brain showed no change in the parasellar mass. Repeat CSF showed an even higher WBC count (2195 leukocytes, 69% neutrophils) and protein (238) with continued hypo-glycorrhachia (26). She underwent an uncomplicated transnasal transsphenoidal surgery for the removal of the sellar lesion (Figure 2 ). Microscopic examination showed a keratin-filled cyst lined with squamous epithelium, typical of epidermoid origin (Figure 3). Symptoms improved and she was discharged on hormone supplement alone. She was asymptomatic at her last follow-up appointment.

## Discussion

This patient presented with an unusual combination of recurrent meningitis and parasellar mass. Dramatic cerebrospinal fluid (CSF) abnormalities included high neutrophil-predominant WBC counts, moderately to markedly increased protein, and low glucose. These findings would usually suggest a bacterial, fungal or tuberculous infection, but an extensive search for these and other organisms was negative. Instead, biopsy showed evidence of a typical epidermoid (sebaceous) cyst. These cysts are thought to cause non-infectious meningeal inflammation through the release of their toxic internal fluid that may contain keratin and fatty acids. They are benign, slow-growing, and typically occur at the skull base (most commonly, the cerebellopontine angle) [1]. They occur predominantly in the pediatric patient population. Epidermoid cysts account for less than 1% of intra-sellar tumors [2]. Most of the studies on epidermoid cysts are case reports which have been presented in pediatric journals. Although multiple intracranial locations have been reported in the past, the sellar origin of epidermoid cysts has not been reported before. The management of these tumors is quite challenging. Clinical and radiological evaluation is critical for a successful surgical outcome. Surgical resection should be followed by the close clinical follow-up to monitor recurrence. Our case emphasizes the importance of keeping a broad differential particularly due to the presentation of a rare pediatric tumor in the adult population. Adult-onset CNS epidermoid cysts in the sella have never been reported before.

## Conclusions

We present a rare case of an adult with recurrent chemical meningitis secondary to a ruptured epidermoid cyst in the sella. Our patient presented with signs and symptoms of meningoencephalitis and was found to have evidence of chemical meningitis on contrast-enhancing sellar mass. Epidermoid cysts have been reported as rare tumors that occur in the pediatric patient population. The intra-sellar location of cysts has never been reported before. Epidermoid cysts are rare tumors that require a multidisciplinary approach including neurologists, radiologists, and surgeons. Early identification and intervention can result in an improved neurological outcome. 

## References

[1] Cherian A, Baheti NN, Easwar HV, Nair DS, Iype T (2012). Recurrent meningitis due to epidermoid. J Pediatr Neurosci.

[2] Velamati R, Hageman JR, Bartlett A (2013). Meningitis secondary to ruptured epidermoid cyst: case-based review. Pediatr Ann.

